# Improvement of bladder function after bladder augmentation surgery: a report of 26 years of clinical experience

**DOI:** 10.1007/s00383-022-05114-1

**Published:** 2022-03-28

**Authors:** Katharina C. Trojan, Joanna Schneider, Beatriz Bañuelos Marco, Luise Ciesla, Tamara Geppert, Angela M. Kaindl, Anja Lingnau

**Affiliations:** 1 Charité – Universitätsmedizin Berlin, Corporate Member of Freie Universität Berlin and Humboldt-Universität zu Berlin, Center of Chronically Sick Children, Augustenburger Platz 1, 13353 Berlin, Germany; 2 Department of Pediatric Neurology, Charité – Universitätsmedizin Berlin, corporate member of Freie Universität Berlin and Humboldt-Universität zu Berlin, Augustenburger Platz 1, 13353 Berlin, Germany; 3 Charité – Universitätsmedizin Berlin, Corporate member of Freie Universität Berlin and Humboldt-Universität zu Berlin, Institute of Cell- and Neurobiology, Charitéplatz 1, 10117 Berlin, Germany; 4 Department of Urology, Charité – Universitätsmedizin Berlin, corporate member of Freie Universität Berlin and Humboldt-Universität zu Berlin, Augustenburger Platz 1, 13353 Berlin, Germany; 5 Berlin Institute of Health at Charité – Universitätsmedizin Berlin, Charitéplatz 1, 10117 Berlin, Germany

**Keywords:** Bladder augmentation, Urodynamic, Spina bifida, Hostility score

## Abstract

**Introduction:**

To assess the long-term effect of bladder augmentation surgery in patients with spina bifida and to identify risk factors for severe bladder dysfunction requiring bladder augmentation.

**Methods:**

A retrospective analysis was performed on 178 patients with spina bifida, 23 of them underwent bladder augmentation. Surgery outcome was evaluated according to urodynamic assessments at three follow-up time points per patient up to 120 months postoperatively. The results were compared to the preoperative situation and to the non-operated control group. Bladder function was evaluated using the modified Hostility score. To identify risk factors for bladder dysfunction requiring bladder augmentation, characteristics such as type of spina bifida, lesion level and therapy of bladder dysfunction were analyzed.

**Results:**

A high spinal lesion level is a risk factor for requiring bladder augmentation. In the BA group, significantly more thoracic lesions were found than NBA group, BA: 26.1%, NBA: 8.4% (*p* = 0.021). With bladder augmentation surgery, the modified Hostility score decreased from a preoperative median value of 4.3 ± 1.4 to 1.6 ± 1.0 at the third postoperative follow-up (FU3 = 61–120 months after surgery). In the reference group, the score of the last urological assessment was 2.0 ± 1.5. The age at which clean intermittent catheterization or anticholinergic medication started had no significant influence on the decision to perform bladder augmentation.

**Discussion/conclusion:**

Spina bifida patients with bladder augmentation had a significant improvement of the bladder function even at long-term follow-up. A high level of spinal lesion was a predisposing factor for requiring a bladder augmentation.

## Introduction

Spina bifida (SB) has an incidence of approximately 4.6:10,000 live births and is associated with a high risk of neurogenic bladder dysfunction [1, 2]. If untreated, the latter can result in renal damage and subsequent chronic kidney disease [3]. Some patients will require surgery to increase bladder capacity and reduce storage pressures. Bladder augmentation is currently the gold standard. This procedure expands the bladder volume; the resulting bladder reservoir protects the upper urinary tract through decrease of the intravesical pressure [4, 5].

Most existing studies focus on one parameter to evaluate the surgery like the perioperative complications, the pre- and postoperative bladder capacity and pressure, or the stage of the vesicoureteral reflux [6–8]. However, to evaluate the bladder function and the renal deterioration risk, parameters such as bladder capacity, intravesical pressure, and vesico-renal reflux need to be collectively evaluated [9].

The aim of this study was to evaluate the efficacy and risk factors for bladder augmentation surgery and to analyze its long-term effects in a cohort of patients with SB. We compared the main characteristics of the augmented group to those of non-augmented patients with SB.

## Methods

A retrospective study was performed on a cohort of 279 patients with SB treated at Charité University Medicine Berlin, between 1990 and 2016. We reviewed medical records of all patients and collected data on medical history, clinical, and radiological findings in a database with standardized variables. Urologic records were available for 178 cases that were subsequently analyzed.

We divided these patients into two groups, BA (bladder augmentation) and NBA (non-bladder augmentation).

We compared the following values in these two groups: gender, type of SB, level of spinal lesion, age at initiation of clean intermittent catheterization, age at first administration of anticholinergic medication and treatment with botulinum-A-toxin.

The anatomic SB lesion level was defined by the first split vertebral arch on spinal MRI scans.

In all patients, hospital policy was to initiate CIC on demand according to clinical or urodynamic findings (e.g. high residual volume, urinary tract infections, demand for anticholinergic medication).

**Fig. 1:**
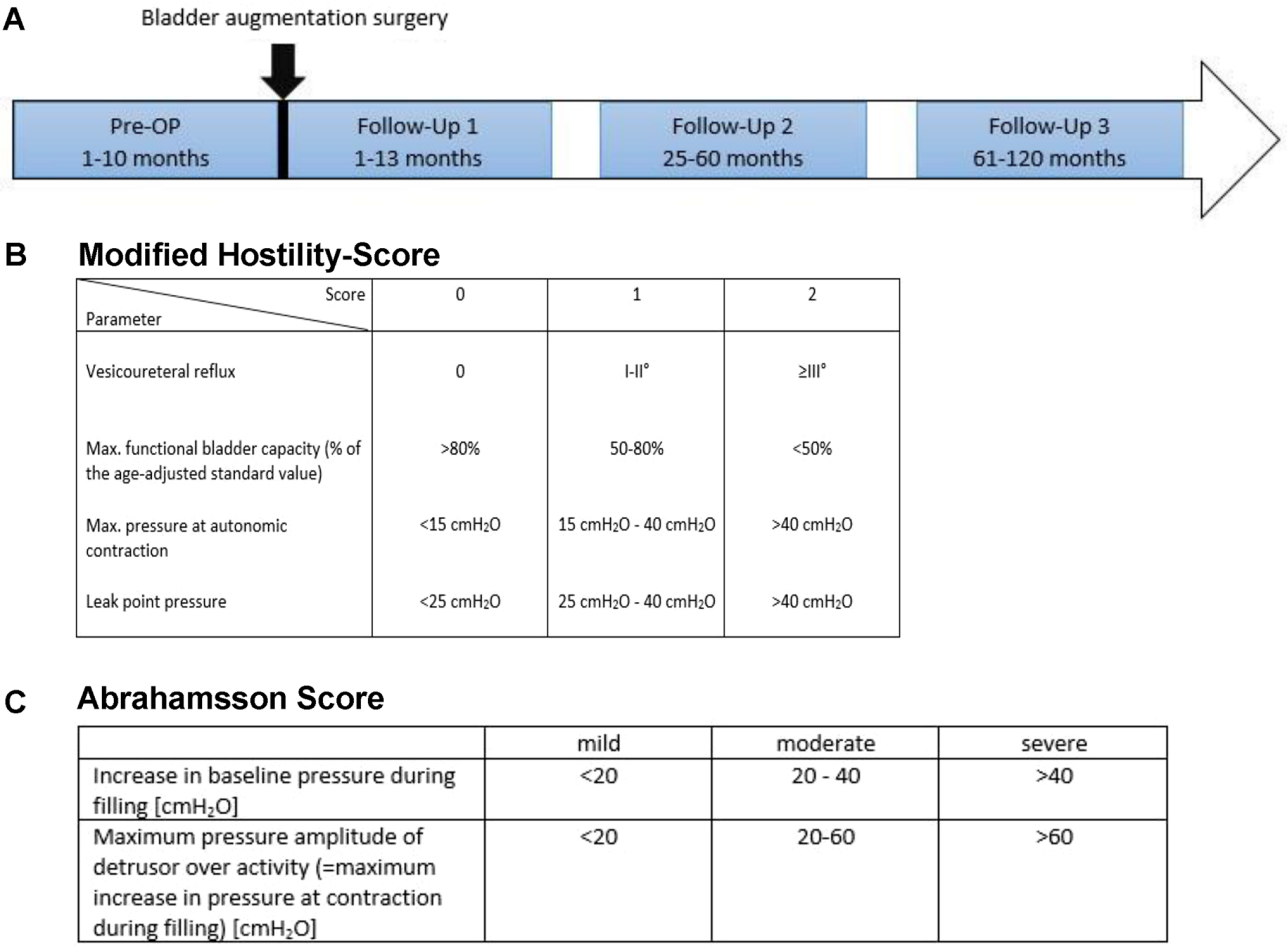
Study design and assessment scores. **A** follow-up-periods, **B** modified Hostility score; **C** Abrahamsson score

In the BA group, the urodynamic results were assessed at four different times: before and after bladder augmentation surgery: 0–10 months before and 1–13, 25–60 and 61–120 months after bladder augmentation surgery. Shown in Fig. 1A.

In case of treatment with botox-A, we chose the closest examination prior to this. In the follow-up, we chose the last available examination. It applied to both groups.

Videourodynamic assessments were performed according to the standards recommended by the International Continence Society (ICS) [10].

For evaluation of the bladder function, we used two different scoring systems, the modified Hostility score [11] and the score reported by Abrahamsson et al. 2007 [12] Shown in Fig. 1B and C.

To compare the continence status at the four different time points, we used the pads per day-test. Continence was defined by no need of pads between clear intermittent catheterization. If a patient needed one to two pads per day, we classified the continence status as mild incontinence/social continence. A usage of more than two pads per 24 h was defined as incontinence.

Statistical analysis and graph design were performed using IBM SPSS Statistics (version 25) and GraphPad Prism (version 7), retrospectively. We used the Shapiro–Wilk test to test the normal distribution of our data, the Pearson’s chi-squared test including Yate’s correction and fisher’s exact test to analyze nominally-scaled variables, the Mann–Whitney *U* test for median comparisons without a normal distribution, the Wilcoxon test to show differences between non-parametric related samples and the Spearman-Rho-test to analyze relations of ordinal-scaled variables. Statistical Significance was defined as *p* < 0.05.

## Results

**Fig. 2:**
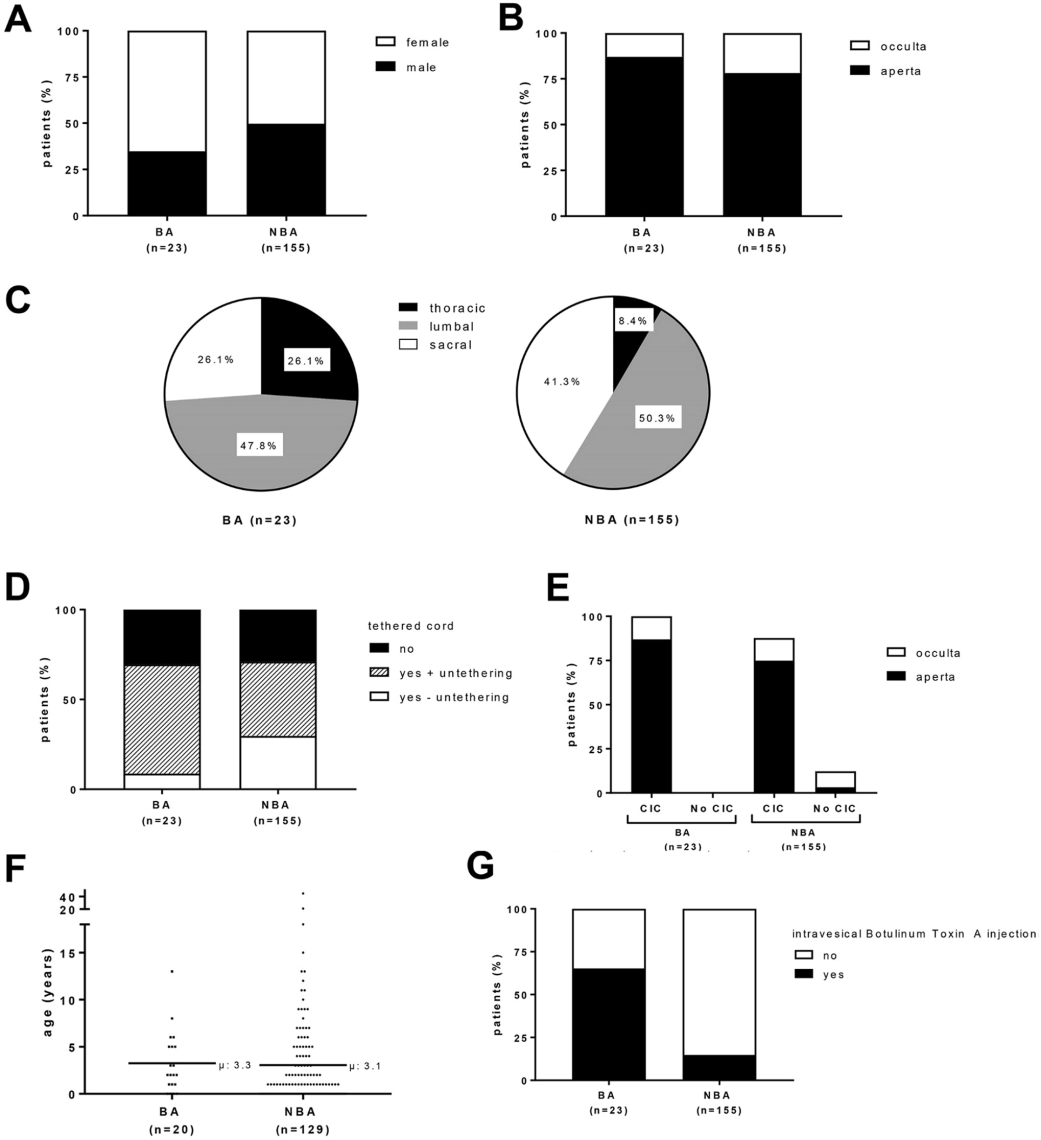
Characteristics of patients with spina bifida who did or did not require bladder augmentation. **A** gender, **B** type of spina bifida, **C** level of the spinal cord lesion, **D** presence of tethered cord, **E** clean intermittent catheterization, **F** age at clean intermittent catheterization initiation, **G**1 intravesical Botulinum Toxin injection

We analyzed urologic data of 178 patients with SB. Gender distribution was almost equal. Shown in Fig. 2A. Most of the patients had spina bifida aperta. Shown in Fig. 2B.

Mean age was 16.1 ± 11.1 years [BA (*n* = 23) 22.2 ± 10.7 years and NBA (*n* = 155) 15.2 ± 10.9 years].

### Level of spinal lesion

Level of the spinal lesion in patients with SB differed between the groups.

Patients in the BA group had significant more thoracic lesions than patients in the NBA group) [BA: 26.1%, NBA: 8.4% (*p* = 0.021)] Shown in Fig. 2C. Sacral lesions were more frequent in the NBA group (sacral: BA: 26.1%, NBA: 41.3%).

Tethered cord was found in 69.6% of the patients receiving BA, 87.5% of them had previously undergone an untethering surgery. In the NBA group, tethered cord was present in 71% of patients, 58.2% of them had previously undergone untethering Shown in Fig. 2D.

### Age at initiation of intermittent catheterization

All patients in the BA group and 87.7% of the NBA group performed clean intermittent catheterization. Shown in Fig. 2E. There was no significant difference in the mean age at initiation of clean intermittent catheterization [BA 3.3 ± 3.3 years; NBA 3.1 ± 5.4 years (*p* > 0.05)]. Shown in Fig. 2F.

Similarly, the two cohorts did not differ significantly in the age at first administration of anticholinergic medication. Patients of the BA group received anticholinergics at 4.4 ± 4.2 years (NBA group 3.4 ± 4.5 years). Treatment with botox-A was prevalent in 65.2% of patients before BA (NBA 14.8%). Shown in Fig. 2G.

### Long-term outcome of the bladder function after bladder augmentation

**Fig. 3:**
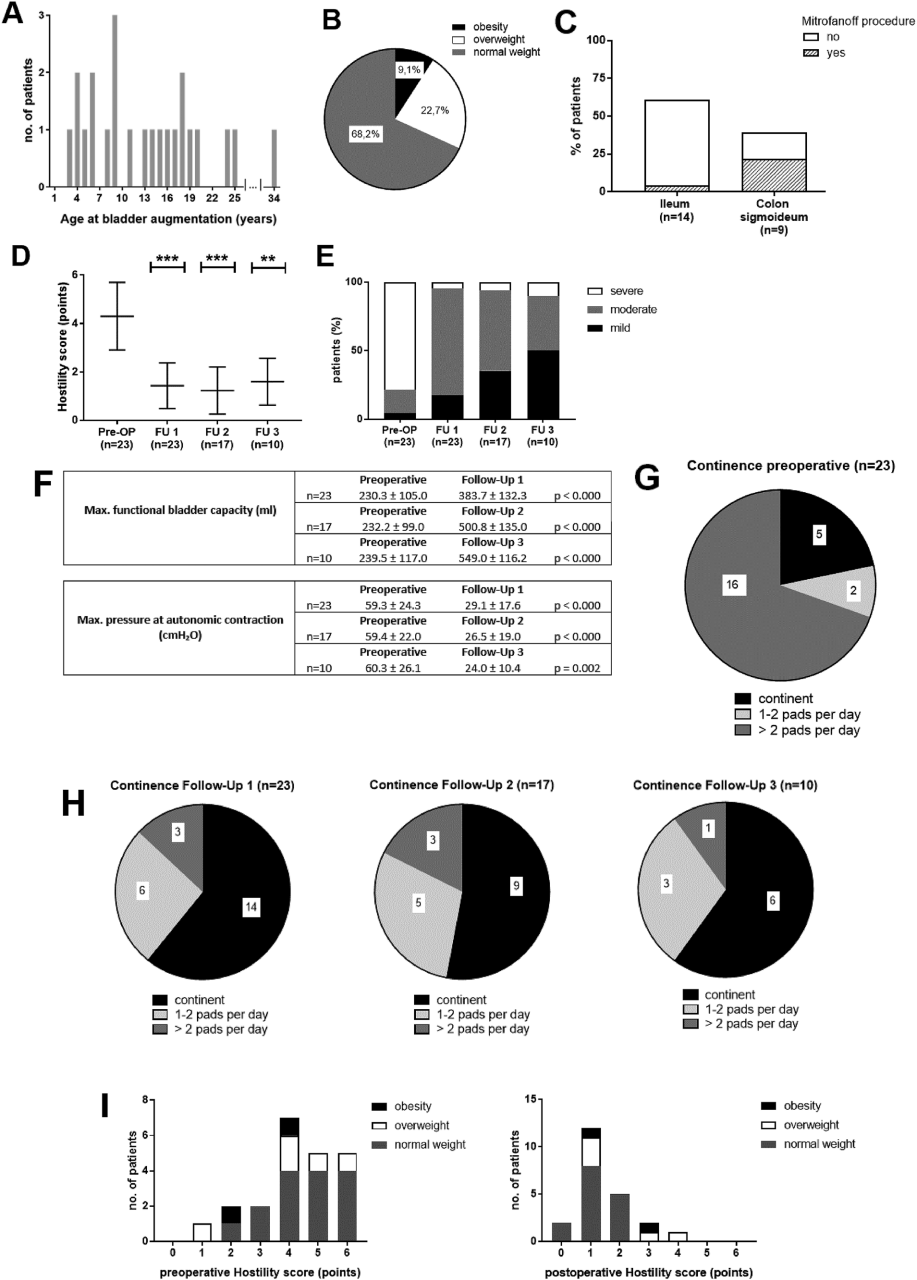
Bladder augmentation surgery. **A** age at surgery, **B** weight at surgery, **C** intestinal segment used for BA, **D** pre- and postoperative modified Hostility score, **E** bladder dysfunction stage pre- and postoperative, **F** pre- and postoperative bladder capacity and maximum pressure at autonomic contraction, **G** preoperative continence, **H** postoperative continence, **I** relation between weight and outcome at the modified Hostility score. Abbreviations: *SB* spina bifida, *BA* bladder augmentation, *NBA* non-bladder augmentation

Mean age at bladder augmentation surgery was 13.4 (± 7.9) years. Shown in Fig. 3A. 21.7% (*n* = 22) were overweight at the time of surgery (90–97 centile for body weight in patients < 18 years or BMI > 25–30 kg/m^2^ in older patients), 8.7% of patients were obese (> 97. centile for body weight in patients < 18 years or BMI > 30 kg/m^2^). Shown in Fig. 3B.

Terminal ileum was used in 60.9% and or colon in 39.1%. Mitrofanoff-stoma placement at the time of BA surgery in 26.1% of patients. Shown in Fig. 3C.

In the BA group, the modified Hostility score decreased significantly after surgery. Shown in Fig. 3D. The modified Hostility score in the reference group was at a median of 2.0 ± 1.5 points. The Abrahamsson score also showed a significant negative tendency in all follow-ups compared to the preoperative situation. Shown in Fig. 3E. Furthermore, we analyzed the development of the bladder capacity and the maximum pressure at autonomic contraction isolated from the two scoring systems, which also showed a significant improvement. Shown in Fig. 3F.

### Continence outcome

Prior to surgery, 16 patients (69.6%) suffered from incontinence defined as a usage of more than two pads per day. Two patients (8.7%) needed one to two pads per 24 h and only five patients were completely continent. Shown in Fig. 3G.

Of these patients, the majority reported an improvement of continence compared to the preoperative situation:

At the time of the first follow-up, 14 patients (60.9%) stated that they were completely continent with no need of pads at all. Six other patients (26.1%) improved to a social continence with a usage of one or two pads per day. Only three patients (13.0%) still suffered from incontinence. After the second and third follow-up, the results maintained, so we can state that continence improved compared to the preoperative situation. Shown in Fig. 3H.

### Influencing factors on the outcome after bladder augmentation surgery

In most patients after BA surgery, the hostility score improved. There were only three patients in this group with a postoperative modified Hostility score above 2 points.

We analyzed the possible causes and found that excess body weight correlated significantly with a worse outcome according to the modified Hostility score (*p* = 0.005). Shown in Fig. 3I.

### Complications

BA-associated complications occurred in five patients. One had twice a bladder perforation (4 months and 2 years postoperatively).

Insufficiency of augmentation was seen in one case, requiring a second BA surgery 6 years after the first one. Two patients suffered from bladder stones (1 and 2 years postoperatively), and one patient had a Mitrofanoff-stoma-revision 12 years after BA surgery due to Mitrofanoff-stoma-leakage.

## Discussion

The aim of this study was to assess the long-term effect and identify risk factors for bladder augmentation surgery in patients with spina bifida.

A predisposing factor for requiring BA surgery was a high (thoracic) level of the spinal lesion. Here, we could show that spina bifida patients with a higher lesion were more at risk for undergoing bladder augmentation surgery due to severe bladder dysfunction.

The latter is in line with a previous report of an association between a high lesion level and bladder reconstruction surgeries [13]. Data from patients with spinal cord injuries show that the expected severity of bladder dysfunction is dependent on the level of the spinal cord injury. Upper motor neuron lesions due to complete injuries proximal to the sacral spinal cord result into detrusor over activity. These patients are also at risk for detrusor-sphincter dyssynergia, resulting in incomplete bladder emptying and/or high bladder pressure [14, 15]. However, this applies to complete lesions. Since spina bifida is failure of fusion of the neuronal tube resulting in incomplete palsy, every patient needs to be evaluated individually and the severity of bladder dysfunction assessed, independent of the level of spinal lesion.

Our results showed that patients who underwent BA surgery had a significant improvement of their bladder function at long-term follow-up. Existing studies included only a little number of patients with this specific disease or focus on only one parameter [8, 16].

In our view, it is important to evaluate different parameters collectively to make a real statement about the bladder function.

Bladder augmentation surgery showed a significant improvement of the bladder function in the long-term outcome up to 10 years after the surgery. Furthermore, the Hostility score of the augmented patients is postoperatively lower when compared to the non-augmented patients.

Another clinical relevant finding is the remarkable improvement of the continence status. Prior to the bladder augmentation surgery, only five patients were continent between clean intermittent catheterization. After the surgery, 14 patients were completely continent and 6 patients were social continent in the first follow-up. For most patients, incontinence has a huge impact on their quality of life [17] 18. Wiener et al. showed a significant association between continence and employment status in patients with spina bifida age 25 years or older [19]. This shows that even an improvement from complete incontinence to social continence (one or two pads per day) can change a patient’s quality of life. In our study, from the 18 patients, who suffered preoperatively from incontinence or social continence, 15 patients improved to a better stadium, which means less or no pads per day, after the surgery. This may be the biggest impact of the surgery on the patients’ lives.

We could show an association of excess weight with a worse bladder function outcome after surgery. However, even in these cases, BA improved the bladder function. Paying attention to the preoperative body weight and possibly reducing weight before surgery may enhance the surgery effect. Donovan et al. already claimed in his study that there is an association between a patient’s body mass index and the risk of complications at urinary or fecal reconstructive procedures [20]. With our data, we can add the fact that obesity is associated with a worse outcome in bladder augmentation surgeries. Nevertheless, overweight should not be seen as an exclusion criterion for BA surgery, especially with the knowledge that obesity is a common comorbidity in patients with spina bifida [21].

Our data also showed that the age at which patients start performing clean intermittent catheterization does not differ between the two groups, which suggests that this is not a risk factor for undergoing BA surgery. Similarly, the age at which patients start taking anticholinergic medication does not appear as a predictive factor. These statements might be surprising with the literature [22–24] and need to be interpreted in context of the fact that CIC as well as anticholinergic medication was in our cohort not initiated from the newborn period but individually according to clinical and urodynamic findings.

Even if we analyzed the data of our big spina bifida center, where many patients with this disease were treated since years, the two sub-cohorts differ a lot in their number of patients. From our experience, the parents of these chronically sick children are reluctant about another big surgery and often try to avoid or stall the decision to perform bladder augmentation surgery. This might also be the reason for the heterogeneous cohort of patients with a wide age range.

Another limitation of our study is the fact that the intestinal segment used for the augmentation was ileum in 14 patients and colon in the other 9 patients. The decision about the surgical technique depends mainly on the surgeon’s choice. We understand that this may bias our results. Due to the retrospective character of this study, standardized quality of life questionnaires were not available for all patients, which would have been is particularly interesting for evaluation of the continence outcome.

Therefore, we need more studies with a higher number of patients and a prospective study design to prevent loss of data. Especially studies, which include parameters to evaluate the kidney function and timing of initiation of CIC would improve the situation of rare literature.

## Conclusion

In summary, bladder augmentation surgery seems to be an effective and long-lasting method for patients who are beyond treatment with conservative methods, to improve their quality of life and to protect the upper urinary tract from damage.

## Data Availability

All data generated or analyzed during this study are included in this article. Further enquiries can be directed to the corresponding author.

## Abbreviations

SB: Spina bifida
BA: Bladder augmentation
NBA: Non-bladder augmentation
